# Molecular detection of vector-borne bacteria in bat ticks (Acari: Ixodidae, Argasidae) from eight countries of the Old and New Worlds

**DOI:** 10.1186/s13071-019-3303-4

**Published:** 2019-01-22

**Authors:** Sándor Hornok, Krisztina Szőke, Marina L. Meli, Attila D. Sándor, Tamás Görföl, Péter Estók, Yuanzhi Wang, Vuong Tan Tu, Dávid Kováts, Sándor A. Boldogh, Alexandra Corduneanu, Kinga M. Sulyok, Miklós Gyuranecz, Jenő Kontschán, Nóra Takács, Ali Halajian, Sara Epis, Regina Hofmann-Lehmann

**Affiliations:** 1 Department of Parasitology and Zoology, University of Veterinary Medicine, Budapest, Hungary; 2 Clinical Laboratory, Department of Clinical Diagnostics and Services, and Center for Clinical Studies, Vetsuisse Faculty, University of Zurich, Zurich, Switzerland; 3 Department of Parasitology and Parasitic Diseases, University of Agricultural Sciences and Veterinary Medicine, Cluj-Napoca, Romania; 4 Department of Zoology, Hungarian Natural History Museum, Budapest, Hungary; 5 Department of Zoology, Eszterházy Károly University, Eger, Hungary; 6 Department of Pathogenic Biology, School of Medicine, Shihezi University, Shihezi, China; 7 Institute of Ecology and Biological Resources, Vietnam Academy of Science and Technology, Hanoi, Vietnam; 8 Hungarian Biodiversity Society, Budapest, Hungary; 9 Directorate, Aggtelek National Park, Jósvafő, Hungary; 10 Institute for Veterinary Medical Research, Centre for Agricultural Research, Hungarian Academy of Sciences, Budapest, Hungary; 11 Plant Protection Institute, Centre for Agricultural Research, Hungarian Academy of Sciences, Budapest, Hungary; 12 Department of Biodiversity, School of Molecular and Life Sciences, Faculty of Science and Agriculture, University of Limpopo, Sovenga, South Africa; 13 Department of Biosciences and Pediatric Clinical Research Center “Romeo and Enrica Invernizzi”, University of Milan, Milan, Italy

**Keywords:** Chiroptera, Soft tick, Hard tick, *Rickettsia*, *Anaplasma*, *Bartonella*, *Haemoplasma*

## Abstract

**Background:**

Despite the increasingly recognized eco-epidemiological significance of bats, data from molecular analyses of vector-borne bacteria in bat ectoparasites are lacking from several regions of the Old and New Worlds.

**Methods:**

During this study, six species of ticks (630 specimens) were collected from bats in Hungary, Romania, Italy, Kenya, South Africa, China, Vietnam and Mexico. DNA was extracted from these ticks and analyzed for vector-borne bacteria with real-time PCRs (screening), as well as conventional PCRs and sequencing (for pathogen identification), based on the amplification of various genetic markers.

**Results:**

In the screening assays, *Rickettsia* DNA was only detected in bat soft ticks, whereas *Anaplasma phagocytophilum* and haemoplasma DNA were present exclusively in hard ticks. *Bartonella* DNA was significantly more frequently amplified from hard ticks than from soft ticks of bats. In addition to *Rickettsia helvetica* detected by a species-specific PCR, sequencing identified four *Rickettsia* species in soft ticks, including a *Rickettsia africae*-like genotype (in association with a bat species, which is not known to migrate to Africa), three haemotropic *Mycoplasma* genotypes in *Ixodes simplex*, and *Bartonella* genotypes in *I. ariadnae* and *I. vespertilionis*.

**Conclusions:**

Rickettsiae (from both the spotted fever and the *R. felis* groups) appear to be associated with soft rather than hard ticks of bats, as opposed to bartonellae. Two tick-borne zoonotic pathogens (*R. helvetica* and *A. phagocytophilum*) have been detected for the first time in bat ticks. The present findings add Asia (China) to the geographical range of *R. lusitaniae*, as well as indicate the occurrence of *R. hoogstraalii* in South Africa. This is also the first molecular evidence for the autochthonous occurrence of a *R. africae*-like genotype in Europe. Bat haemoplasmas, which are closely related to haemoplasmas previously identified in bats in Spain and to “*Candidatus* Mycoplasma haemohominis”, are reported here for the first time from Central Europe and from any bat tick.

**Electronic supplementary material:**

The online version of this article (10.1186/s13071-019-3303-4) contains supplementary material, which is available to authorized users.

## Background

Bats (order Chiroptera) are the only mammals which actively fly. Among the consequences of this trait, bats show a geographically widespread distribution and may even undergo short to long distance seasonal migration [1]. Additionally, the evolution of flight in bats yielded inadvertent consequences on their immune functioning, and therefore bats are special in their capacity to act as reservoir hosts for intracellular pathogens [2]. Bats frequently reach high population densities in or near urban habitats, and their ticks may blood-feed on humans [3, 4], which further increases their veterinary-medical importance.

The presence of DNA from vector-borne bacteria in bat ticks appears to be most extensively studied in Europe. In western Europe, *Rickettsia* and *Ehrlichia* species have been molecularly identified in soft ticks (*Argas vespertilionis*) of bats (in France [5] and the UK [6]). Another study carried out in central Europe (Poland) failed to detect *Borrelia burgdorferi* (*s.l.*), rickettsiae and *Anaplasma phagocytophilum* in the bat-associated hard tick species, *Ixodes vespertilionis* [7]. Nonetheless, literature data on molecular analyses of vector-borne bacteria in bat ticks are lacking from several regions of the Old and New Worlds. Therefore, during this study, bat ticks collected in countries representing less-studied regions (eastern and southern Europe, central and southeast Asia, eastern Africa, central America) were screened for the presence of DNA from four important genera of vector-borne bacteria, which include zoonotic species.

## Methods

DNA extracts of 307 hard ticks (*I. ariadnae*: 26 larvae, 14 nymphs, 5 females; *I*. *vespertilionis*: 89 larvae, 27 nymphs, 8 females; *I*. *simplex*: 79 larvae, 50 nymphs, 9 females) and 323 soft ticks (*A. vespertilionis*: 321 larvae; *A. transgariepinus*: 1 larva; *Ornithodoros* sp.: 1 larva) were used. The hard ticks (Acari: Ixodidae) were collected from 200 individuals of 17 bat species in two countries (Hungary, Romania), whereas soft ticks (Acari: Argasidae) were removed from 59 individuals of 17 bat species in eight countries (Hungary, Romania, Italy, Kenya, South Africa, China, Vietnam and Mexico) [8, 9]. The geographical coordinates and/or locations of collection sites, along with identification of bat and tick species by expert taxonomists (authoring this study), have already been reported [8, 9]. DNA was extracted individually from hard ticks, and individually or in pools of 2–3 specimens (if collected from the same host individual) from soft ticks, as reported [8, 9].

Bat tick DNA extracts (*n* = 514) were screened for the presence of *Rickettsia helvetica*, other *Rickettsia* spp., *A. phagocytophilum*, haemotropic *Mycoplasma* spp. and *Bartonella* spp. with real-time PCRs (Additional file 1: Table S1). This was followed by conventional PCRs and sequencing of various genetic markers (Additional file 2: Table S2), and phylogenetic analyses (Additional file 3: Text S1) except for *R. helvetica* and *A. phagocytophilum*.

Prevalences were compared with Fisherʼs exact test.

## Results and discussion

*Rickettsia* DNA was only detected in bat soft ticks (all three evaluated species), whereas *Anaplasma phagocytophilum* and three haemotropic *Mycoplasma* genotypes were present exclusively in the hard tick species *I. simplex* (Table 1). In addition, *Bartonella* DNA was significantly more frequently detected in hard than in soft ticks of bats (Fisherʼs exact test: *P* = 0.01).

**Table 1:** Prevalence of pathogen DNA in bat ticks according to bat host species and country of origin. The latter are referred to with superscript letters (the cumulative number of bat individuals is equal to or less than the number of positives, because one or more ticks could have been collected from a single bat). After the name of the tick species, the number of analyzed DNA extracts is shown, which corresponds to the number of tick individuals (except for *A. vespertilionis*, in the case of which pooled samples were also used)

	Soft ticks			Hard ticks		
	*A. vespertilionis* (*n* = 205)	*A. transgariepinus* (*n* = 1)	*Ornithodoros* sp. (*n* = 1)	*I. vespertilionis* (*n* = 124)	*I. ariadnae* (*n* = 45)	*I. simplex* (*n* = 138)
*Rickettsia* spp.	120^a^/205 (58.5%)	1^b^/1 (100%)	1^c^/1 (100%)	–	–	–
*Anaplasma phagocytophilum*	–	–	–	–	–	2^d^/138 (1.4%)
*Bartonella* spp.	2^e^/205 (1%)	–	–	5^f^/124 (4%)	5^g^/45 (11.1 %)	6^h^/138 (4.3%)
Haemoplasmas	–	–	–	–	–	1^i^/138 (0.7%)

^a^*Pipistrellus pipistrellus* (Hungary 6×, Italy 1×); *Pi. pygmaeus* (Hungary 10×); *Pi. nathusii* (Hungary 1×); *Pi. kuhlii* (Hungary 1×); *Pi. abramus* (Vietnam 1×); *Pi.* cf. *rueppellii* (Kenya 1×); *Myotis brandtii* (Hungary 1×); *My. alcathoe* (Hungary 2×); *My. dasycneme* (Hungary 5×); *Plecotus auritus* (Hungary 1×); *Pl. austriacus* (Hungary 3×); *Nyctalus noctula* (Hungary 1×); *Eptesicus serotinus* (Hungary 1×, Romania 1×); *Vespertilio murinus* (Hungary 2×, China 1×)

^b^*Pi. hesperidus* (South Africa 1×)

^c^*Balantiopteryx plicata* (Mexico 1×)

^d^*Miniopterus schreibersii* (Hungary 1×, Romania 1×)

^e^*Pi. pygmaeus* (Hungary 2×)

^f^*My. daubentonii* (Romania 2×); *My. capaccinii* (Romania 1×); *Eptesicus serotinus* (Romania 1×); *Rhinolophus ferrumequinum* (Romania 1×)

^g^*My. alcathoe* (Hungary 1×); *My. bechsteinii* (Hungary 1×); *My. daubentonii* (Hungary 3×)

^h^*Mi. schreibersii* (Romania 5×)

^i^*Mi. schreibersii* (Hungary 1×)

In particular, *R. helvetica* was identified in one soft tick (*A. vespertilionis*) from China. This finding is consistent with former reports of *R. helvetica* in bat fleas [10] and bat faeces [11] in Hungary. Taking into account the bat host-specificity of these PCR-positive ectoparasites, it is possible that bats are susceptible to *R. helvetica*, although based on the very low prevalence this may have low epidemiological significance.

In four samples of *A. vespertilionis* from Hungary, the same *Rickettsia* genotype was identified, which was reported from bat soft ticks collected in France (GenBank: JN038177, see Table 2) [12]. More importantly, in one *A. vespertilionis* from Hungary rickettsial DNA was detected, which in the amplified part of the *gltA* gene had 99.9–100% sequence identity (depending on the nucleotide at position 679: C or T) to sequences of *R. africae* from Ethiopia (GenBank: CP001612) and from migratory bird fleas reported in neighboring Slovakia (GenBank: HM538186) [13]. Two other markers were also successfully amplified from this sample: the *17 kDa* gene sequence was identical with that of several *Rickettsia* species, whereas the *OmpA* sequence showed 2 bp differences from that of *R. africae* (Table 2).

**Table 2:** Results of molecular analyses and sequence comparisons. Species names of rickettsiae are based on highest sequence similarities to *gltA* sequences available on GenBank and published in peer-reviewed papers

Genotype/species	Country (no. of positive samples)	Highest sequence similarity in GenBank shown as gene: bp/bp (%)	Closest match sequence accession number	Accession number (this study)	Reference
*Rickettsia helvetica*	China (1)	–	–	–	–
*Rickettsia* sp. Av22	Hungary (4)	*gltA*: 757/757 (100)	JN038177	MH383138	Socolovschi et al. [5]
		*17 kDa*: 394/394 (100)	several	MH383143	–
		*OmpA*: 477/477 (100)	several	MH383147	–
*Rickettsia africae*-like	Hungary (1)	*gltA*: 757/757 (100)	CP001612	MH383139	Sekeyová et al. [12]
		*17 kDa*: 394/394 (100)	several	MH383144	–
		*OmpA*: 475/477 (99.6)	CP001612	MH383148	Sekeyová et al. [12]
*Rickettsia hoogstraalii*	South Africa (1)	*gltA*: 757/757 (100)	FJ767737	MH383140	Duh et al. [17]
		*17 kDa*: 390/390 (100)^a^	FJ767736	MH383145	Duh et al. [17]
*Rickettsia lusitaniae*	Mexico (1)	*gltA*: 757/757 (100)^b^	JQ771933	MH383141	Milhano et al. [18]
	China (2)	*gltA*: 756/757 (99.9)	JQ771933	MH383142	Milhano et al. [18]
		*17 kDa*: 393/394 (99.7)	JQ771934	MH383146	Milhano et al. [18]
		*OmpA*: 461/464 (99.4)	JQ771935	MH383149	Milhano et al. [18]
*Anaplasma phagocytophilum*	Hungary (1)	–	–	–	–
	Romania (1)	–	–	–	–
*Bartonella* sp. Ia23	Hungary (1)	*gltA*: 313/317 (98.7)	KX300154	MH544201	Urushadze et al. [20]
		ITS: 520/529 (98.3)^c^	MF288126	MH544202	McKee et al. [21]
*Bartonella* sp. Iv76	Romania (1)	*gltA*: 317/317 (100)	KR822802	MH578453	Lilley et al. [22]
		ITS: 291/306 (95.1)	MF288124	MH544203	McKee et al. [21]
*Mycoplasma* sp. Is128-1	Hungary (1)	*16S* rRNA: 953/954 (99.9)	KM538692	MH383150	Millán et al. [23]
*Mycoplasma* sp. Is128-2	Hungary (1)	*16S* rRNA: 824/826 (99.8)	KM538698	MH383151	Millán et al. [23]
*Mycoplasma* sp. Is128-3	Hungary (1)	*16S* rRNA: 952/954 (99.8)	KM538692	MH383152	Millán et al. [23]

*Rickettsia helvetica* and *Anaplasma phagocytophilum* were detected by using species-specific primers (Additional file 1: Table S1) and sequencing was not possible due to high Ct values

^a^Amplification of *OmpA* gene was not successful

^b^Amplifications of *17 kDa* and *OmpA* genes were not successful

^c^Amplification of the *ftsZ* gene was not successful

**Fig. 1:**
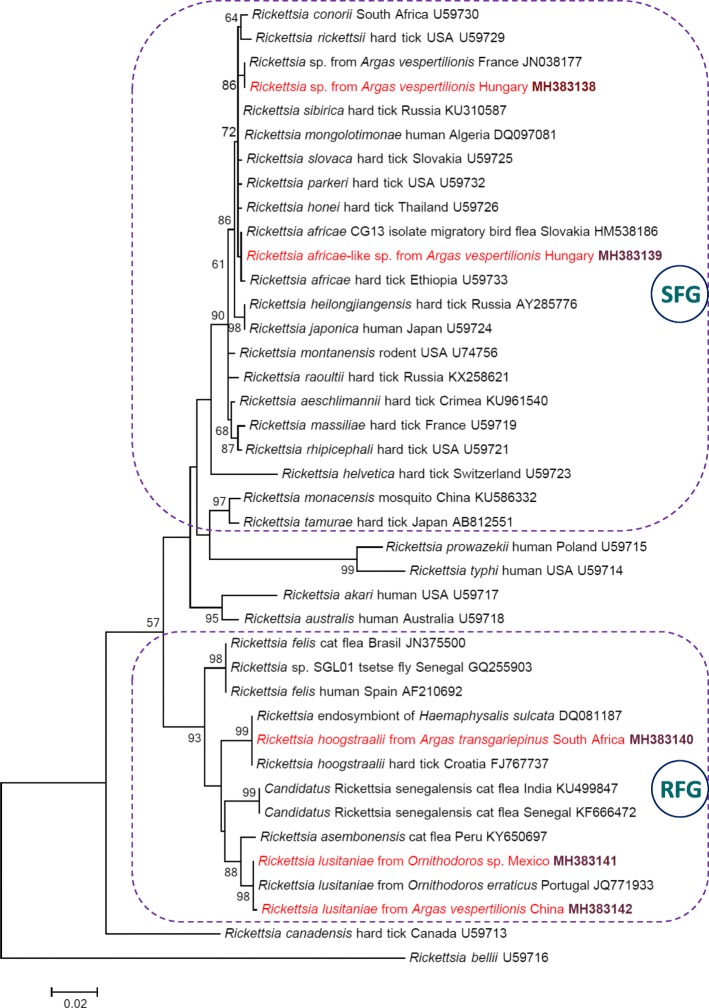
Maximum-likelihood tree of spotted fever group (SFG: encircled with dashed line), *Rickettsia felis* group (RFG: encircled with dashed line) and other rickettsiae based on the *gltA* gene. Sequences from this study are highlighted with red color and bold accession numbers. Branch lengths represent the number of substitutions per site inferred according to the scale shown

Interestingly, the *OmpA* sequence from this *A. vespertilionis* was identical with that of the *Rickettsia* strain “Atlantic rainforest” (GenBank: MF536975 [14]) and *Rickettsia* sp. “Atlantic rainforest Aa46” (GenBank: KY113110 [15]), which represent a genetic variant of the human pathogen *R. parkeri* [14, 15] detected so far only in the New World. Nevertheless, we consider the species detected in *A. vespertilionis* to belong to *R. africae* because of the following four reasons: (i) the *gltA* gene is a reliable genetic marker for species identification and phylogenetic comparison of rickettsiae [13, 16]; (ii) *R. africae* was identified based on this gene in previous studies (e.g. [13]); (iii) the *gltA* phylogenetic analysis confirmed that the rickettsial genotype from *A. vespertilionis* collected in Hungary clustered with *R. africae*, but apart from *R. parkeri* (Fig. 1); and (iv) the *OmpA* gene of the type strain of *R. parkeri* (GenBank: U43802) was only 98.3% (469/477 bp) identical with the *OmpA* sequence obtained here.

The soft tick containing the *R. africae*-like DNA was collected from *Myotis dasycneme*, which occurs north of the Mediterranean Basin and is a facultative, middle distance migrant bat species, not known to move between Europe and Africa [1]. Therefore, this result implies the autochthonous occurrence of a *R. africae*-like genotype in Europe. In the phylogenetic analysis, this genotype was clearly separated (with moderate, 72% bootstrap support value) from the *Rickettsia* sp. from *A. vespertilionis* reported in France (Fig. 1).

In addition, *R. hoogstraalii* was identified in a soft tick from South Africa (Table 2). This rickettsia has only been reported from Europe and North America [17], therefore its occurrence in Africa is new. Similarly, *R. lusitaniae* was formerly only reported in Europe (Portugal) [18] and Central America (Mexico) [19], the latter being confirmed in the present study (Table 2). However, a *gltA* genotype highly similar to *R. lusitaniae* (1 bp difference from JQ771933, i.e. 99.9% identity) was also shown here, for the first time, to occur in Asia (China) (Table 2). The level of *OmpA* sequence divergence of this Chinese isolate (MH383149) was the same (3 bp) from *R. lusitaniae* in Portugal (JQ771935) and from *R. lusitaniae* in Mexico (GenBank: KX377432).

In summary, bat soft ticks contained the DNA of three *Rickettsia* species from the spotted fever group (SFG), and two further ones from the *Rickettsia felis* group (RFG) (Fig. 1).

*Anaplasma phagocytophilum* DNA was detected here in the hard tick species, *I. simplex*, in both Hungary and Romania. Previously, *Anaplasma* sp. DNA was also shown to be present in bat feces in Hungary (GenBank: KP862895). This low prevalence in bat ticks, suggests that bats may be susceptible to this pathogen, but most likely play a subordinate (if any) role in the epidemiology of granulocytic anaplasmosis in the evaluated region.

Bartonellae associated with bat ectoparasites, including ticks, have been reported for the first time in Hungary [10]. Based on high Ct values of the majority of bartonella-positive samples here, sequencing was only possible from two hard ticks (one *I. ariadnae* and one *I. vespertilionis*; Table 2). Based on two genetic markers (*gltA* and ITS), *Bartonella* sp. “Ia23” from *I. ariadnae* was relatively (Table 2: 98.2–98.7%) similar to *Bartonella* sp. isolates detected in bats (*My. emarginatus*) in Georgia, Caucasus [20, 21]. In *I. vespertilionis*, known to feed on humans [3], *Bartonella* sp. “Iv76” was shown to be present (Table 2). The *gltA* sequence of this genotype was 100% (317/317 bp) identical to “*Candidatus* Bartonella hemsundetiensis”, reported from Finland [22] (GenBank: KR822802, Table 2), but only 99.7% (316/317 bp) identical to *Bartonella* sp. isolates (GenBank: KX300127, KX300131, KX300136) detected in bats (*My. blythii*) in Georgia, Caucasus [20]. The ITS sequence of *Bartonella* sp. “Iv76” was 95.1% (291/306 bp) and 93.8% (287/306 bp) identical to *Bartonella* sp. isolates (GenBank: MF288124 and KX420717, respectively) from bats (*My. blythii* and *My. emarginatus*, respectively) sampled in Georgia, Caucasus [21]. The *ftsZ* sequence similarity of *Bartonella* sp. “Iv76” (GenBank: MH544204) to bat-associated bartonellae available on GenBank from Georgia [20] was below 85.5% (data not shown).

**Fig. 2:**
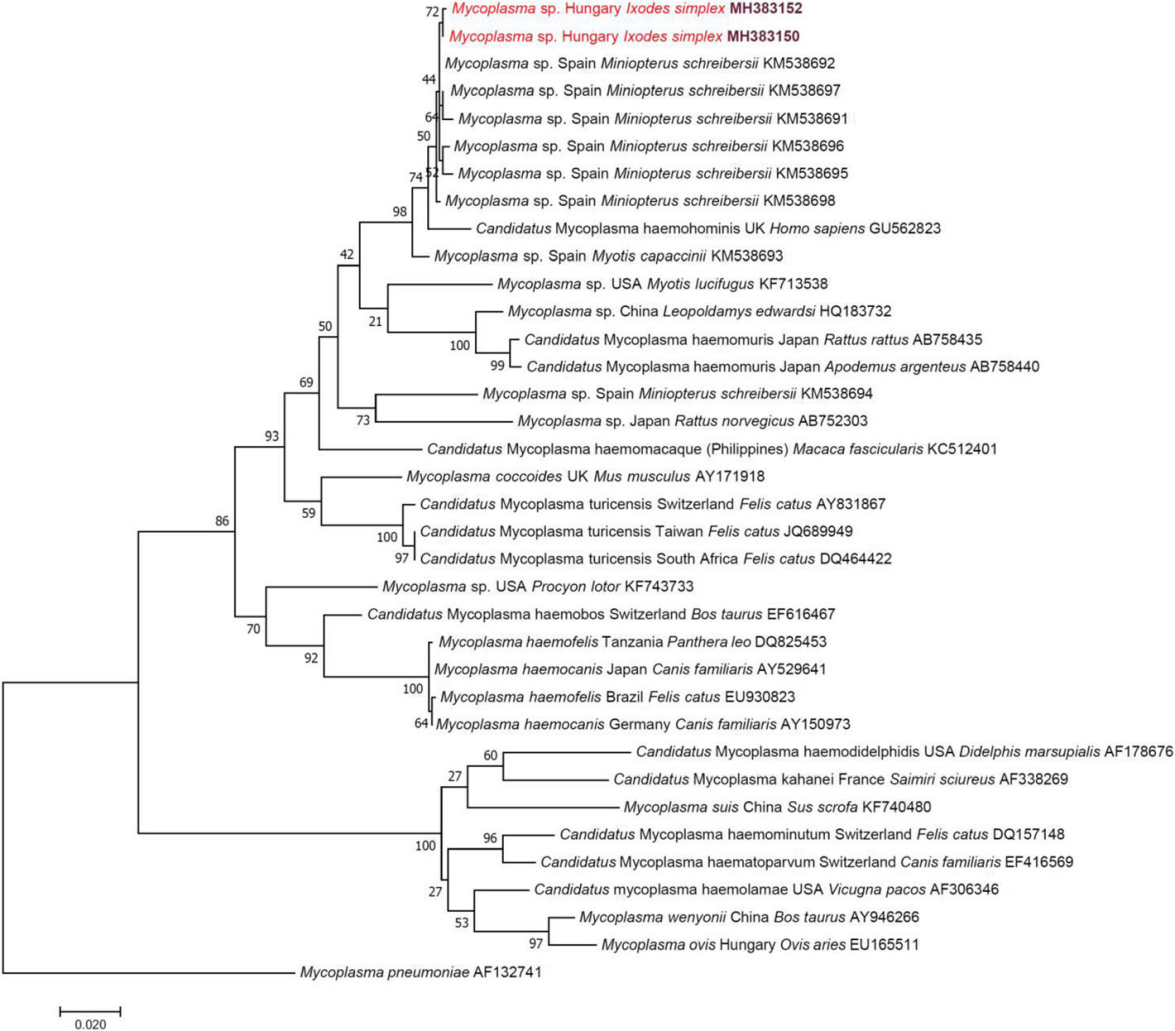
Maximum-likelihood tree of haemotropic *Mycoplasma* spp. based on the *16S* rRNA gene. Sequences from this study are highlighted with red color and bold accession numbers. After the country name, the isolation source is indicated with genus and species name. Branch lengths represent the number of substitutions per site inferred according to the scale shown

In Europe, molecular evidence on the occurrence of bat haemoplasmas has hitherto been reported from western countries, i.e. Spain [23] and the Netherlands [11]. Based on blood and fecal samples, respectively, these studies suggested infections of bats with the relevant agents. Haemoplasmas are regarded as predominantly vector-borne [24]. However, bat-associated haemoplasmas have not hitherto been identified in blood-sucking arthropods. Here, three haemotropic *Mycoplasma* genotypes have been detected in a tick specimen (*I. simplex*), collected in Hungary (Table 2). *Ixodes simplex* is specialized to its host, *Miniopterus schreibersii* [25], from which bat species haemoplasma genotypes having 99.8–99.9% *16S* rRNA gene similarity to those from *I. simplex* collected in Hungary (Table 2) have been reported in Spain [23]. Importantly, these bat-associated haemoplasmas are phylogenetically close to “*Candidatus* Mycoplasma haemohominis”, as reported [23] and as also shown here (Fig. 2).

## Conclusions

Rickettsiae (from both the spotted fever and the *R. felis* groups) appear to be associated with soft rather than hard ticks of bats, as opposed to bartonellae. Although with low prevalence, two tick-borne zoonotic pathogens (*R. helvetica* and *A. phagocytophilum*) have been detected for the first time in bat ticks. The present findings add Asia (China) to the geographical range of *R. lusitaniae*, as well as indicate the occurrence of *R. hoogstraalii* in South Africa. This is also the first molecular evidence of a *R. africae*-like genotype in Europe, in association with a bat host species that is not known to migrate to Africa. Bat haemoplasmas, which are phylogenetically close to “*Ca.* M. haemohominis”, are reported here for the first time from central Europe and from any bat tick.

## Additional files

Additional file 1: **Table S1.** Technical data for real-time PCRs used for screening. (DOCX 18 kb)

Additional file 2: **Table S2.** Technical data for conventional PCRs used for sequencing. (DOCX 21 kb)

Additional file 3: **Text S1.** Methods. (DOCX 20 kb)

## Abbreviations

Ct: Threshold cycle
ftsZ: Cell division protein
gltA: Citrate synthase
ITS: 16S*-*23S rRNA intergenic spacer region
OmpA: Outer membrane protein-A
