# Supplementary material for: Molecular detection of vector-borne bacteria in bat ticks (Acari: Ixodidae, Argasidae) from eight countries of the Old and New Worlds
Source: Parasit Vectors. 2019 Jan 22;12:50. doi: 10.1186/s13071-019-3303-4 (PMC6343265; doi:10.1186/s13071-019-3303-4)
Supplement: Supplementary file 1 — Table S1. Technical data for real-time PCRs used for screening. (DOCX 18 kb) [file 13071_2019_3303_MOESM1_ESM.docx]

**Additional file 1: Table S1.** Technical data for real-time PCRs used for screening.

| **Target pathogen categories**  **(target gene)** | **Oligonucleotides** (sequence 5'-3') | **Reference for original method (modified protocol)** |
| --- | --- | --- |
| *Rickettsia helvetica*  (*23S rRNA*) | Rickhelv.147f (TTT GAA GGA GAC ACG GAA CAC A)  Rickhelv.211r (TCC GGT ACT CAA ATC CTC ACG TA)  Rickhelv.170p (6-FAM-AAC CGT AGC GTA CAC TTA-MGBNFQ) | Boretti et al. [1] |
| other rickettsiae  (*gltA*) | CS-F (TCG CAA ATG TTC ACG GTA CTT T)  CS-R (TCG TGC ATT TCT TTC CAT TGT G)  CS-P (6-FAM-TGC AAT AGC AAG AAC CGT AGG CTG GAT G-BHQ-1) | Boretti et al. [1] |
| *Anaplasma phagocytophilum*  (*Msp2*) | ApMSP2f (ATG GAA GGT AGT GTT GGT TAT GGT ATT)  ApMSP2r (TTG GTC TTG AAG CGC TCG TA)  ApMSP2p (TGG TGC CAG GGT TGA GCT TGA GAT TG-HEX) | Courtney et al. [2]  (Hornok et al. [3]) |
| haemoplasmas  (*16S rRNA*) | Sybr_For (AGC AAT RCC ATG TGA ACG ATG AA)  Sybr_Rev1 (TGG CAC ATA GTT TGC TGT CAC TT)  Sybr_Rev2 (GCT GGC ACA TAG TTA GCT GTC ACT) | Willi et al. [4]  (Hornok et al. [5]) |
| *Bartonella* spp.  (*gltA*) | Bart.738f (GGT GCT AAT CCA TTT GCA TGT ATT)  Bart.831r (GTA ACA TTT TTA GGC ATG CTT CAT TA)  Bart.772p (6-FAM-AGC TGG TCC CCA AAG GCA TGC AA-TAMRA) | Molia et al. [6] |

**References**

1. Boretti FS, Perreten A, Meli ML, Cattori V, Willi B, Wengi N, et al. [Molecular investigations of *Rickettsia helvetica* infection in dogs, foxes, humans and *Ixodes* spp. ticks.](http://www.biomedexperts.com/Abstract.bme/16490318/Canine_neosporosis_in_Hungary_screening_for_seroconversion_of_household_herding_and_stray_dogs) Appl Environ Microbiol. 2009;75:3230–7.
2. Courtney JW, Kostelnik LM, Zeidner NS, Massung RF. Multiplex real-time PCR for detection of *Anaplasma phagocytophilum* and *Borrelia burgdorferi*. J Clin Microbiol. 2004;42,3164–3168.
3. [Hornok S](https://www.ncbi.nlm.nih.gov/pubmed/?term=Hornok%20S%5BAuthor%5D&cauthor=true&cauthor_uid=25127161), [Meli ML](https://www.ncbi.nlm.nih.gov/pubmed/?term=Meli%20ML%5BAuthor%5D&cauthor=true&cauthor_uid=25127161), [Gönczi E](https://www.ncbi.nlm.nih.gov/pubmed/?term=G%C3%B6nczi%20E%5BAuthor%5D&cauthor=true&cauthor_uid=25127161), [Halász E](https://www.ncbi.nlm.nih.gov/pubmed/?term=Hal%C3%A1sz%20E%5BAuthor%5D&cauthor=true&cauthor_uid=25127161), [Takács N](https://www.ncbi.nlm.nih.gov/pubmed/?term=Tak%C3%A1cs%20N%5BAuthor%5D&cauthor=true&cauthor_uid=25127161), [Farkas R](https://www.ncbi.nlm.nih.gov/pubmed/?term=Farkas%20R%5BAuthor%5D&cauthor=true&cauthor_uid=25127161), et al. Occurrence of ticks and prevalence of *Anaplasma phagocytophilum* and *Borrelia burgdorferi* s.l. in three types of urban biotopes: forests, parks and cemeteries. [Ticks Tick Borne Dis.](https://www.ncbi.nlm.nih.gov/pubmed/25127161) 2014;5:785–9.
4. [Willi B](https://www.ncbi.nlm.nih.gov/pubmed/?term=Willi%20B%5BAuthor%5D&cauthor=true&cauthor_uid=19828748), [Meli ML](https://www.ncbi.nlm.nih.gov/pubmed/?term=Meli%20ML%5BAuthor%5D&cauthor=true&cauthor_uid=19828748), [Lüthy R](https://www.ncbi.nlm.nih.gov/pubmed/?term=L%C3%BCthy%20R%5BAuthor%5D&cauthor=true&cauthor_uid=19828748), [Honegger H](https://www.ncbi.nlm.nih.gov/pubmed/?term=Honegger%20H%5BAuthor%5D&cauthor=true&cauthor_uid=19828748), [Wengi N](https://www.ncbi.nlm.nih.gov/pubmed/?term=Wengi%20N%5BAuthor%5D&cauthor=true&cauthor_uid=19828748), [Hoelzle LE](https://www.ncbi.nlm.nih.gov/pubmed/?term=Hoelzle%20LE%5BAuthor%5D&cauthor=true&cauthor_uid=19828748), et al. Development and application of a universal Hemoplasma screening assay based on the SYBR green PCR principle. [J Clin Microbiol.](https://www.ncbi.nlm.nih.gov/pubmed/?term=willi+sybr+principle) 2009;47:4049–54.
5. [Hornok S](https://www.ncbi.nlm.nih.gov/pubmed/?term=Hornok%20S%5BAuthor%5D&cauthor=true&cauthor_uid=25248165), [Abichu G](https://www.ncbi.nlm.nih.gov/pubmed/?term=Abichu%20G%5BAuthor%5D&cauthor=true&cauthor_uid=25248165), [Meli ML](https://www.ncbi.nlm.nih.gov/pubmed/?term=Meli%20ML%5BAuthor%5D&cauthor=true&cauthor_uid=25248165), [Tánczos B](https://www.ncbi.nlm.nih.gov/pubmed/?term=T%C3%A1nczos%20B%5BAuthor%5D&cauthor=true&cauthor_uid=25248165), [Sulyok KM](https://www.ncbi.nlm.nih.gov/pubmed/?term=Sulyok%20KM%5BAuthor%5D&cauthor=true&cauthor_uid=25248165), [Gyuranecz M](https://www.ncbi.nlm.nih.gov/pubmed/?term=Gyuranecz%20M%5BAuthor%5D&cauthor=true&cauthor_uid=25248165), et al. Influence of the biotope on the tick infestation of cattle and on the tick-borne pathogen repertoire of cattle ticks in Ethiopia. [PLoS One.](https://www.ncbi.nlm.nih.gov/pubmed/?term=hornok+ethiopia+biotope) 2014;9:e106452.
6. Molia S, Chomel BB, Kasten RW, Leutenegger CM, Steele BR, Marker L, et al. Prevalence of *Bartonella* infection in wild African lions (*Panthera leo*) and cheetahs (*Acinonyx jubatus*). Vet Microbiol. 2004;100:31–41.
