# Supplementary material for: Molecular detection of vector-borne bacteria in bat ticks (Acari: Ixodidae, Argasidae) from eight countries of the Old and New Worlds
Source: Parasit Vectors. 2019 Jan 22;12:50. doi: 10.1186/s13071-019-3303-4 (PMC6343265; doi:10.1186/s13071-019-3303-4)
Supplement: Supplementary file 2 — Table S2. Technical data for conventional PCRs used for sequencing. (DOCX 21 kb) [file 13071_2019_3303_MOESM2_ESM.docx]

**Additional file 2: Table S2.** Technical data for conventional PCRs used for sequencing.

| **Target group in this study (family / *genus*)** | **Gene (~ amplicon length)** | **Forward and reverse primers (5' - 3') (Reference)** | **Temperature and duration of:** | | | | | **Number of cycles** |
| --- | --- | --- | --- | --- | --- | --- | --- | --- |
|  |  |  | **Initial denaturation** | **Denaturation** | **Annealing** | **Extension** | **Final extension** |  |
| *Rickettsia* spp. | *gltA* (798 bp) | CS477f (GGA AGC AGA CTA CGA ACT TA)  CS1273r (GAT AAC CAG TGT AAA GCT GT)  (Sekeyová et al. [1]) | 98°C, 3m | 98°C, 10s | 56°C, 30s | 72°C, 30s | 72°C, 5m | 35/45 |
| *Rickettsia* spp. | *17 kDa* protein (434 bp) | 17kD1 (GCT CTT GCA ACT TCT ATG TT)  17kD2 (CAT TGT TCG TCA GGT TGG CG)  (Williams et al. [2]) | 98°C, 3m | 98°C, 10s | 56°C, 30s | 72°C, 1m | 72°C, 10m | 40 |
| *Rickettsia* spp. | *OmpA* (533 bp) | Rr190.70p (ATG GCG AAT ATT TCT CCA AAA)  Rr190.602n (AGT GCA GCA TTC GCT CCC CCT)  (Regnery et al. [3]) | 95°C, 5m | 95°C, 20s | 48°C, 30s | 72°C, 1m | 72°C, 5m | 40 |
| haemoplasmas | *16S rRNA* (871 bp) | HemMycop16S-41s (GYA TGC MTA AYA CAT GCA AGT CGA RCG)  HemMyco16S-938as (CTC CAC CAC TTG TTC AGG TCC CCG TC)  (Mascarelli et al. [4]) | 98°C, 3m | 98°C, 1m | 63°C, 30s | 72°C, 2m | 72°C, 10m | 45 |
|  | *16S rRNA*  (1030 bp) | HemMycop16S-322s (GCC CAT ATT CCT ACG GGA AGC AGC AGT)  HemMycop16S-1420as (GTT TGA CGG GCG GTG TGT ACA AGA CC)  (Mascarelli et al. [4]) | 98°C, 3m | 98°C, 1m | 68°C, 30s | 72°C, 2m | 72°C, 10m | 45 |
| *Bartonella* spp. | *ftsZ* (935 bp) | Bfp11 (ATT AAT CTG CAC CGG CCA GA)  Bfp12 (ATT AAT CTG CAT CGG CCA GA)  Bsp2w (ACV GAD ACA CGA ATA ACA CC)  (Zeaiter et al. [5]) | 98°C, 3m | 98°C, 10s | 56°C, 30s | 72°C, 1m | 72°C, 10m | 45 |
| *Bartonella* spp. | *16S-23S rRNA* ITS (≤600 bp) | 325s (CTT CAG ATG ATG ATC CCA AGC CTT CTG GCG)  1100as (GAA CCG ACG ACC CCC TGC TTG CAA AGC A)  (Diniz et al. [6]) | 95°C, 5m | 94°C, 30s | 65°C, 30s | 72°C, 50s | 72°C, 5m | 40 |
| *Bartonella* spp. | *gltA* (359 bp) | CSH1f (GCG AAT GAA GCG TGC CTA AA )  BhCS.1137n (AAT GCA AAA AGA ACA GTA AAC A)  (Kamani et al. [7]) | 98°C, 3m | 98°C, 10s | 56°C, 30s | 72°C, 30s | 72°C, 10m | 35 |

**Abbreviations**: m=minutes, s=seconds.

**References**

1. Sekeyová Z, Fournier PE, Rehacek J, Raoult D. Characterization of a new spotted fever group rickettsia detected in *Ixodes ricinus* (Acari: Ixodidae) collected in Slovakia. Med Entomol. 2000;37:707–13.
2. Williams SG, Sacci JB, Schriefer ME, Anderson EM, Fujioka KK, Sorvillo FJ, et al. Typhus and typhus-like rickettsiae associated with opossums and their fleas in Los Angeles county, California. J Clin Microbiol. 1992;30:1758–62.
3. Regnery RL, Spruill CL, Plikaytis BD. Genotypic identification of Rickettsiae and estimation of interspecies sequence divergence for portions of two rickettsial genes. J Bacteriol. 1991;173:1576–89.
4. Mascarelli PE, Keel MK, Yabsley M, Last LA, Breitschwerdt EB, Maggi RG. Hemotropic mycoplasmas in little brown bats (*Myotis lucifugus*). Parasit Vectors. 2014; 7:117.
5. [Zeaiter Z](https://www.ncbi.nlm.nih.gov/pubmed/?term=Zeaiter%20Z%5BAuthor%5D&cauthor=true&cauthor_uid=12354859), [Liang Z](https://www.ncbi.nlm.nih.gov/pubmed/?term=Liang%20Z%5BAuthor%5D&cauthor=true&cauthor_uid=12354859), [Raoult D](https://www.ncbi.nlm.nih.gov/pubmed/?term=Raoult%20D%5BAuthor%5D&cauthor=true&cauthor_uid=12354859). Genetic classification and differentiation of *Bartonella* species based on comparison of partial ftsZ gene sequences. [J Clin Microbiol.](https://www.ncbi.nlm.nih.gov/pubmed/?term=zeaiter+Genetic+Classification+and+Differentiation+of+Bartonella+Species+Based+on+Comparison+of+Partial+ftsZ+Gene+Sequences) 2002;40:3641–7.
6. [Diniz PP](https://www.ncbi.nlm.nih.gov/pubmed/?term=Diniz%20PP%5BAuthor%5D&cauthor=true&cauthor_uid=17583666), [Maggi RG](https://www.ncbi.nlm.nih.gov/pubmed/?term=Maggi%20RG%5BAuthor%5D&cauthor=true&cauthor_uid=17583666), [Schwartz DS](https://www.ncbi.nlm.nih.gov/pubmed/?term=Schwartz%20DS%5BAuthor%5D&cauthor=true&cauthor_uid=17583666), [Cadenas MB](https://www.ncbi.nlm.nih.gov/pubmed/?term=Cadenas%20MB%5BAuthor%5D&cauthor=true&cauthor_uid=17583666), [Bradley JM](https://www.ncbi.nlm.nih.gov/pubmed/?term=Bradley%20JM%5BAuthor%5D&cauthor=true&cauthor_uid=17583666), [Hegarty B](https://www.ncbi.nlm.nih.gov/pubmed/?term=Hegarty%20B%5BAuthor%5D&cauthor=true&cauthor_uid=17583666), [Breitschwerdt EB](https://www.ncbi.nlm.nih.gov/pubmed/?term=Breitschwerdt%20EB%5BAuthor%5D&cauthor=true&cauthor_uid=17583666). Canine bartonellosis: serological and molecular prevalence in Brazil and evidence of co-infection with *Bartonella henselae* and *Bartonella vinsonii* subsp. berkhoffii. [Vet Res.](https://www.ncbi.nlm.nih.gov/pubmed/17583666) 2007;38:697–710.
7. Kamani J, Baneth G, Mitchell M, Mumcuoglu KY, Gutiérrez R, Harrus S. *Bartonella* species in bats (Chiroptera) and bat flies (Nycteribiidae) from Nigeria, West Africa. Vector Borne Zoonotic Dis. 2014;14:625–32.
