# Supplementary material for: Molecular detection of vector-borne bacteria in bat ticks (Acari: Ixodidae, Argasidae) from eight countries of the Old and New Worlds
Source: Parasit Vectors. 2019 Jan 22;12:50. doi: 10.1186/s13071-019-3303-4 (PMC6343265; doi:10.1186/s13071-019-3303-4)
Supplement: Supplementary file 3 — Text S1. Methods. (DOCX 20 kb) [file 13071_2019_3303_MOESM3_ESM.docx]

**Additional file 3: Methods**

**Real-time PCRs used for screening**

All tests were run with all samples at the Clinical Laboratory of Vetsuisse Faculty, University of Zurich, Switzerland (except the screening for *Anaplasma phagocytophilum*). These methods are summarized in Table 1.

**Conventional PCRs used for sequencing**

These methods are summarized in Table 2. Sequencing was attempted with a selected number of samples according to their results in the screening assays (i.e., for rickettsiae: n = 9, Ct < 28; for haemoplasmas: n = 1; and for bartonellae: n = 2, Ct < 39). The tests were performed with the following reaction mixture components.

For the amplification of the citrate synthase (*gltA*) gene of *Rickettsia* spp., the cell division protein (*ftsZ*) gene of *Bartonella* spp. and the *gltA* gene of *Bartonella* spp. the reaction mixture contained 5 µl of 5× Phusion HF buffer (Finnzymes, Espoo, Finland), 0.5 µl of 2U/µl Phusion Hot Start DNA polymerase (Finnzymes), 0.5 µl (final concentrations of 0.2 mM) dNTPs (Sigma-Aldrich, Buchs, Switzerland), 0.625 µl (final concentration: 0.5 µM) of each primer (Microsynth, Balgach, Switzerland), 15.25 µl nuclease-free water and 2.5 µl of template DNA in a final volume of 25 µl.

For the amplification of the *17kDa* surface antigen (*htrA*) gene of *Rickettsia* spp. the reaction mixture contained 10 µl of 5× Phusion HF buffer (Finnzymes), 0.5 µl of 2U/µl Phusion Hot Start DNA polymerase (Finnzymes), 1 µl (final concentrations of 0.2 mM) dNTPs (Sigma-Aldrich), 1.25 µl (final concentration: 0.5 µM) of each primer (Microsynth), 31 µl nuclease-free water and 5 µl of template DNA in a final volume of 50 µl.

The above tests were performed at the Clinical Laboratory (Vetsuisse Faculty, University of Zurich, Switzerland). PCR products were visualized in a 1.5% or 2% agarose gel, depending on the size of PCR product.

In addition, for amplification of the outer membrane protein-A (*OmpA*) gene of *Rickettsia* spp. and of the *16S-23S rRNA* intergenic spacer region (ITS) of *Bartonella* spp., the reaction mixture (25 μl) contained 1 U (0.2 μl) HotStarTaq Plus DNA polymerase, 2.5 μl 10× CoralLoad Reaction buffer (including 15 mM MgCl_2_), 0.5 μl PCR nucleotide Mix (0.2 mM each), 0.5 μl (1 μM final concentration) of each primer, 15.8 μl ddH_2_O and 5 μl template DNA.

The latter two tests were performed at the Department of Parasitology and Zoology (University of Veterinary Medicine, Hungary). PCR products were visualized in 1.5% agarose gel. Purification and Sanger dideoxy sequencing were done by Microsynth GmbH (Balgach, Switzerland) or Biomi Inc. (Gödöllő, Hungary), depending on where the PCRs were performed.

**Cloning and sequencing of haemoplasmas**

PCR products were purified with the QIAquick Gel Extraction Kit (QIAGEN®, Hilden, Germany) according to the manufacturer’s instructions. After the addition of 3’ A-overhangs, purified PCR products were cloned into the pCR™II-TOPO® TA vector (Thermo Fisher Scientific Inc.). Plasmid DNA was purified from 5 colonies using a QIAprep Spin Miniprep kit (Qiagen). Inserts were sequenced using the M13 forward and reverse primers (Microsynth).

**Phylogenetic and statistical analyses**

Sequences were aligned and compared to GenBank data by nucleotide BLASTN program (https://blast.ncbi.nlm.nih.gov). Representative sequences were submitted to GenBank (accession numbers: Table 2). The MEGA model selection method was applied to choose the appropriate model for phylogenetic analyses. For the phylogenetic analyses, sequences with high coverage were retrieved from GenBank and trimmed to the same length. This dataset was resampled 1,000 times to generate bootstrap values. Phylogenetic analyses were conducted with the Maximum Likelihood method and Tamura-3 model by using MEGA version 7.0. Prevalence rates were compared with Fisher's exact test, and the limit of significance was set to 0.05.
